# 注射用盐酸美法仑预处理自体造血干细胞移植治疗多发性骨髓瘤125例的疗效和安全性

**DOI:** 10.3760/cma.j.issn.0253-2727.2023.02.011

**Published:** 2023-02

**Authors:** 文阳 黄, 薇 刘, 慧敏 刘, 燕 徐, 齐 王, 辰星 杜, 文捷 熊, 伟薇 隋, 菲 田, 静 王, 树华 易, 刚 安, 录贵 邱, 德慧 邹

**Affiliations:** 中国医学科学院北京协和医学院血液病医院（中国医学科学院血液学研究所），实验血液学国家重点实验室，国家血液系统疾病临床医学研究中心，细胞生态海河实验室，天津 300020 State Key Laboratory of Experimental Hematology, National Clinical Research Center for Blood Diseases, Haihe Laboratory of Cell Ecosystem, Institute of Hematology & Blood Diseases Hospital, Chinese Academy of Medical Sciences & Peking Union Medical College, Tianjin 300020, China

大剂量美法仑预处理自体造血干细胞移植（auto-HSCT）是年轻、无严重脏器功能障碍的多发性骨髓瘤（MM）患者的一线标准治疗策略[1]–[3]。传统的静脉美法仑制剂使用丙二醇作为助溶剂，溶解度较低，稳定性较差，且有发生丙二醇相关不良反应的风险[4]。注射用盐酸美法仑（商品名迈维宁^®^）使用特殊改良的环糊精为溶剂，使药物的可溶性及稳定性得以明显改善，也避免了丙二醇相关不良反应的发生[5]–[7]。该药于2019年8月在我国正式上市。本研究对在我中心接受盐酸美法仑200 mg/m^2^预处理auto-HSCT的125例MM患者进行回顾性分析，评估其疗效和安全性。

## 病例与方法

1. 病例：2019年8月至2021年9月于中国医学科学院血液病医院接受诱导治疗序贯auto-HSCT的125例MM患者纳入本研究。纳入标准：①年龄≤70岁；②无严重脏器功能障碍，内生肌酐清除率≥40 ml/min；③诱导治疗后获得≥部分缓解（PR）治疗反应；④接受盐酸美法仑200 mg/m^2^预处理；⑤移植后随访超过100 d。所有患者均符合国际骨髓瘤工作组（IMWG）诊断标准[8]。MM分期采用国际分期系统（ISS分期）和2015年修订的国际分期系统（R-ISS）[9]。

2. 自体外周血造血干细胞动员和预处理方案：自体外周血造血干细胞动员方案采用化疗联合重组人粒细胞集落刺激因子（rhG-CSF）±普乐沙福（Plerixafor）或单纯rhG-CSF±普乐沙福。所有患者均以单药大剂量盐酸美法仑作为移植预处理：移植前3 d（−3 d）和−2 d静脉输注盐酸美法仑100 mg·m^−2^·d^−1^（约3 h输毕）。输注盐酸美法仑前15 min至输注结束后30 min期间内，患者口腔含冰以预防黏膜炎。移植后24～48 h给予单剂聚乙二醇化重组人粒细胞刺激因子（PEG-rhG-CSF）6 mg皮下注射或移植后5 d开始予以rhG-CSF 5～10 µg·kg^−1^·d^−1^皮下注射，直至中性粒细胞绝对计数（ANC）超过0.5×10^9^/L并持续2 d。

3. 定义及疗效评估标准：移植相关死亡（TRM）定义为auto-HSCT后100 d内发生的非复发/进展的死亡。根据美国国家癌症研究所不良事件常用术语标准（CTCAE）5.0版评估不良反应；采用世界卫生组织（WHO）口腔毒性反应量表评估口腔黏膜炎。

通过每日血常规结果评估骨髓细胞清除以及中性粒细胞和血小板植入时间。清髓性造血抑制定义为ANC<0.5×10^9^/L或淋巴细胞绝对计数<0.1×10^9^/L或血小板计数<20×10^9^/L。中性粒细胞植入定义为ANC≥0.5×10^9^/L且持续3 d；血小板植入定义为PLT≥20×10^9^/L持续7 d且脱离血小板输注。

MM疗效评价采用IMWG疗效标准[10]，包括完全缓解（CR）、非常好的部分缓解（VGPR）、部分缓解（PR）、疾病稳定（SD）和疾病进展（PD）。

4. 随访：通过查阅患者住院/门诊病历和电话随访方式获得患者随访资料和信息。随访截止时间为2022年1月31日。移植后中位随访12（4～28）个月。

## 结果

1. 临床特征：男65例，女60例，中位年龄57（33～70）岁。IgG型62例（49.6％），IgA型24例（19.2％），IgD型8例（6.4％），轻链型25例（20.0％），不分泌型6例（4.8％）。ISS分期：Ⅰ期22例（17.6％），Ⅱ期58例（46.4％），Ⅲ期45例（36.0％）。R-ISS分期：Ⅰ期17例（13.6％），Ⅱ期81例（64.8％），Ⅲ期27例（21.6％）。125例MM患者均为一线诱导治疗获得≥PR疗效后序贯auto-HSCT。中位诱导疗程数为4（3～8）个，均采用含蛋白酶体抑制剂（硼替佐米、伊沙佐米）和（或）免疫调节剂（来那度胺、沙利度胺）的三药或四药联合方案。全组病例auto-HSCT前疾病状态：CR 66例（52.8％），VGPR 32例（25.6％），PR 27例（21.6％）。

2. 造血重建和安全性评估：所有患者均顺利完成盐酸美法仑输注，未发生明显输注反应。回输CD34^+^细胞中位数为2.89（1.38～19.18）×10^6^/kg。中性粒细胞、血小板植入的中位时间分别为11（8～38）d、13（9～58）d。所有患者均获得清髓性造血抑制。4级中性粒细胞减少、血小板减少的发生率分别为100％、98.4％，3级贫血的发生率为17.6％（22/125），未发生4级贫血。89.6％的患者发生感染，其中最常见的为发热性中性粒细胞减少症（54.4％，68/125），其他感染包括血流感染（14.4％，8/125）、肺感染（7.2％，9/125）和肠道感染（6.4％，8/125）等，无4级感染发生。所有感染获得治愈。非血液学不良反应主要为消化系统不良反应，包括恶心（125例，100％）、呕吐（100例，80.0％）、黏膜炎（125例，100％）和腹泻（125例，100％）等（表1）。未发生4级不良反应，3级不良反应为腹泻（21例，16.8％）、口腔黏膜炎（7例，5.6％）和呕吐（3例，2.4％）。未发生心脏、肾脏及中枢神经系统不良反应。移植后100 d内移植相关死亡发生率为0。

**表1:** 125例多发性骨髓瘤患者接受盐酸美法仑预处理自体造血干细胞移植的消化道不良反应发生情况［例次（％）］

不良反应	1级	2级	3级	4级	5级
恶心	77（61.6）	48（38.4）	0	0	0
呕吐	68（54.4）	29（23.2）	3（2.4）	0	0
口腔黏膜炎	97（77.6）	21（16.8）	7（5.6）	0	0
腹泻	56（44.8）	48（38.4）	21（16.8）	0	0

3. auto-HSCT后100 d疗效评估：所有患者auto-HSCT后均随访超过100 d。移植后100 d时疗效评估：CR 92例（73.6％），VGPR 20例（16.0％），PR 11例（8.8％），复发/进展2例（1.6％）。37例患者在auto-HSCT后获得疗效进步，其中2例由PR转为CR，12例由PR转为VGPR，23例由VGPR转为CR。

## 讨论

随着新药的不断涌现，MM的疗效得到显著提高，但auto-HSCT目前仍然被认为是适合移植、新诊断MM患者的标准治疗策略，美法仑200 mg/m^2^被推荐为标准预处理方案[1]–[3]。传统冻干美法仑制剂应用丙二醇作为助溶剂，溶解不充分且当大剂量应用或延长暴露时间可致丙二醇相关不良反应（肾功能损伤、心律失常、脓毒症样综合征、高渗血症和阴离子间隙增高的代谢性酸中毒等）[4]。另一方面，传统的静脉美法仑制剂复溶后稳定性差，需要在溶解后1 h内完成输注，也增加了患者短时间内大量输液的潜在风险和临床应用的不便。

盐酸美法仑采用新型磺丁基-β-环糊精（BSES）包合技术，无直接损伤肾功能作用，同时显著提高了美法仑的溶解性和稳定性。Singh等[5]研究显示浓度分别为0.45、1.0、2.0、5.0 g/L盐酸美法仑的稳定性较含丙二醇静脉美法仑分别提高5、9、15、29倍。复溶后的5.0 g/L盐酸美法仑在25 °C室温下1 h或2 °C～8 °C冷藏保存24 h未见明显降解，随后以0.25～5.0 g/L盐水稀释后保存于输液袋中3～29 h仍保持稳定。Aljitawi等[6]的Ⅱa期开放、随机、交叉设计生物等效性研究，对比盐酸美法仑和美法仑传统（爱克兰）的药代动力学。患者第一天先接受半量预处理总剂量的爱克兰，随后第二天接受另一半剂量的盐酸美法仑。药代动力学分析显示盐酸美法仑的最大血浆浓度（Cmax）和血浆浓度-时间曲线下面积（AUC）均较爱克兰高出约10％。Hari等[7]进行的多中心、Ⅱb期临床试验纳入61例中位年龄为62（32～73）岁的MM患者，以盐酸美法仑100 mg·m^−2^·d^−1^×2 d作为预处理方案，无输注反应发生，所有患者均达到清髓目标；中性粒细胞、血小板的中位植入时间分别为12 d、13 d。移植后100 d的TRM为0，ORR为100％，部分患者缓解程度得到进一步加深，3级黏膜炎、口腔炎的发生率分别为10％、5％，无4级黏膜炎和口腔炎发生。该研究证实了MM患者接受一线或挽救性大剂量盐酸美法仑预处理auto-HSCT的安全性和有效性。本组125例患者应用盐酸美法仑预处理方案，患者均获得清髓性造血抑制，未发生移植相关死亡，中性粒细胞、血小板的中位植入时间分别为11 d、13 d，3级消化道不良反应发生率分别为为腹泻（16.8％）、口腔黏膜炎（5.6％）和呕吐（2.4％），未发生4级消化道不良反应。移植后100 d评价疗效，37例（29.6％）患者获得疗效进步。

近期两个临床研究对比了盐酸美法仑、爱克兰预处理方案治疗MM的安全性和疗效。Miller等[11]比较了在美国Mayo诊所分别应用盐酸美法仑（216例）和爱克兰（200例）预处理auto-HSCT的MM患者，结果显示两组的住院天数、发热性中性粒细胞减少症、静脉格拉司琼应用、≥2级口腔/食道黏膜炎、静脉液体输注、麻醉镇痛药物使用等指标均无差别。盐酸美法仑组腹泻的发生率较高（82％对71％，*P*＝0.015），但大多数并非艰难梭状芽胞杆菌感染（5％对11％，*P*＝0.038）。两组移植后100 d血液学反应和无进展生存期相似。Monahan等[12]的研究纳入连续294例MM患者，丙二醇静脉美法仑、盐酸美法仑预处理分别为162、132例，盐酸美法仑组的中位年龄更大且平均Karnofsky评分更低。应用倾向加权分析显示盐酸美法仑组中性粒细胞植入时间较短（12 d对13 d，*P*<0.001），移植后30 d内3/4级感染（1.5％对8.0％，*P*＝0.048）和出院后再住院（6.8％对17.9％，*P*＝0.040）的发生率较低，两组间黏膜炎、器官毒性、治疗反应和移植后100 d移植相关死亡率无差异。

综上，本研究结果显示。MM患者诱导治疗获得≥PR后序贯盐酸美法仑200 mg/m^2^预处理auto-HSCT可进一步提高疗效且具有良好的安全性，盐酸美法仑可作为传统静脉美法仑的替代选择。
